# Adequacy of the examination-based licensing system and a training-based licensing system for midwifery license according to changes in childbirth medical infrastructure in Korea: a survey-based descriptive study

**DOI:** 10.3352/jeehp.2023.20.15

**Published:** 2023-05-22

**Authors:** Yun Mi Kim, Sun Hee Lee, Sun Ok Lee, Mi Young An, Bu Youn Kim, Jum Mi Park

**Affiliations:** 1 College of Nursing, Gachon University, Incheon, Korea; 2 Department of Nursing, Gimcheon University, Gimcheon, Korea; 3 Department of Nursing, Silla University, Pusan, Korea; 4 Department of Nursing, Yeoju Institute of Technology, Yeoju, Korea; 5 College of Nursing, Ewha Womans University, Seoul, Korea; 6 Department of Nursing, Namseoul University, Cheonan, Korea; Hallym University, Korea

**Keywords:** Health personnel, Licensure, Midwifery, Republic of Korea, Surveys and questionnaires

## Abstract

**Purpose:**

The number of Korean midwifery licensing examination applicants has steadily decreased due to the low birth rate and lack of training institutions for midwives. This study aimed to evaluate the adequacy of the examination-based licensing system and the possibility of a training-based licensing system.

**Methods:**

A survey questionnaire was developed and dispatched to 230 professionals from December 28, 2022 to January 13, 2023, through an online form using Google Surveys. Descriptive statistics were used to analyze the results.

**Results:**

Responses from 217 persons (94.3%) were analyzed after excluding incomplete responses. Out of the 217 participants, 198 (91.2%) agreed with maintaining the current examination-based licensing system; 94 (43.3%) agreed with implementing a training-based licensing system to cover the examination costs due to the decreasing number of applicants; 132 (60.8%) agreed with establishing a midwifery education evaluation center for a training-based licensing system; 163 (75.1%) said that the quality of midwifery might be lowered if midwives were produced only by a training-based licensing system, and 197 (90.8%) said that the training of midwives as birth support personnel should be promoted in Korea.

**Conclusion:**

Favorable results were reported for the examination-based licensing system; however, if a training-based licensing system is implemented, it will be necessary to establish a midwifery education evaluation center to manage the quality of midwives. As the annual number of candidates for the Korean midwifery licensing examination has been approximately 10 in recent years, it is necessary to consider more actively granting midwifery licenses through a training-based licensing system.

## Graphical abstract

[Fig f1-jeehp-20-15]

## Introduction

### Background/rationale

Korea is currently experiencing a low birth rate, with a total birth rate of 0.81 persons in 2021 (versus the Organization of Economic Co-operation and Development average of 1.61) and 260,562 births [1]. Furthermore, maternal aging is a major issue, the rate of cesarean section performance is 55.8 % [2], and the infant mortality rate is 2.4 per 1,000 births. The maternal mortality rate during birth has fluctuated (9.9 in 2019, 11.8 in 2020, and 8.8 persons in 2021 per 100,000 births) [3,4]. In addition to the very low birth rate and the issue of maternal aging, childbirth care in Korea has been in a critical condition due to low birth rates and low medical fees for deliveries. Meanwhile, in the countries that experienced low birth rates first, including Japan, the natural birth rate was increased by promoting natural births led by midwives while lowering the rate of cesarean section [5,6]. Korea anticipates showing its ability to cope with the challenging condition of the infrastructure of obstetricians concerning childbirth. Fostering high-quality midwives is essential for this to be carried out successfully [7,8].

In order to foster high-quality midwives, the licensing examination is an essential step to guarantee the minimum knowledge and skills of candidates who have completed specialized training courses to perform their jobs. However, only around 10 candidates for midwifery have applied every year, showing a major difference from other professionals’ licensing examinations in terms of their scale and operation method.

### Objectives

This study aimed to assess the adequacy of the examination-based licensing system for qualified midwives when there are around 10 candidates per year. Specifically, the opinions of midwifery-related professionals were analyzed regarding the maintenance of the current examination-based licensing system; implementation of a training-based licensing system; establishment of a midwifery education evaluation center for a training-based licensing system; the quality of midwifery if a training-based licensing system is applied; and, the promotion of the training of midwives as birth support personnel.

## Methods

### Ethics statement

This study was approved by the Institutional Review Board of Gachon University (approval no., 1041449-202301-HR-001).

### Study design

This was a descriptive study.

### Setting

The study was conducted using an online survey method between December 28, 2022 and January 13, 2023. Participants were not required to provide any identifying information and were not offered any incentives for their involvement.

### Participants

The targeted participants were midwives, nurses, nursing school professors, lecturers, obstetricians, and pediatricians working in Korea. The invited midwives were 868 persons who had received continuing education from the Korea Midwifery Association. Nursing professors, nurses, obstetricians, gynecologists, and pediatricians were identified by convenience sampling among the subjects nationwide. Of them, 230 persons who consented and responded to an online survey were included. Responses from 217 individuals, excluding data with insufficient responses, were included in the analysis. Of 868 midwives, 53 were included in the final analysis. There were no exclusion criteria.

### Variables

The questionnaire included 5 items as variables.

### Data sources/measurement

The questionnaire was developed through a discussion between 6 professors of women’s health nursing, 1 professor of child nursing, 1 professor of community nursing, 2 active midwives, 1 doctoral midwife in the department of women’s health nursing, 1 obstetrician, and 1 pediatrician to validate the content and the relevant details. The survey questionnaire is available from Supplement 1. No reliability test was done because this measurement tool was not based on a Likert scale. Response data from participants are available in Dataset 1.

### Bias

There may have been selection bias since only participants who accepted the invitation email were included.

### Study size

Sample size estimation was not done. All subjects who agreed and responded by email were included.

### Statistical methods

The collected data were analyzed by descriptive statistics, including the frequency, percentage, mean, and standard deviation for data relevant, using IBM SPSS ver. 26.0 (IBM Corp.)

## Results

### Participants

The general demographic characteristics of the subjects are shown in Table 1.

### Licensing examination system vs. training-based licensing system

The survey results on the licensing system for the national examination- and training-based license system are shown in Table 2.

Out of 217 respondents, 198 (91.2%) agreed with maintaining the existing examination-based licensing system, while 123 (56.7%) did not agree with implementing a training-based licensing system. However, 132 (60.8%) agreed to the new establishment of a midwifery education evaluation center to help with the training-based licensing system, 163 (75.1%) responded that the quality of midwives would be lower if they became qualified only through the training-based licensing system, and 197 (90.8%) responded that the training of midwives as birth support personnel should be promoted in Korea.

## Discussion

### Key results

Under the current social atmosphere where childbirth medical infrastructure is in a critical condition, this study showed that the examination-based licensing system is still necessary. However, if a training-based licensing system is implemented, it will be necessary to establish a midwifery education evaluation center to manage the quality of midwives.

### Interpretation

This study revealed that 91.2% (198 persons) agreed with maintaining the existing licensing system involving national examinations. It is currently impossible to transition to a training-based licensing system since those who have completed 1 year of training at a corresponding training institute are eligible to take the licensing examination. Most other countries are conducting a national examination-based licensing system for midwifery [9].

In addition, the results of this study indicate that many professionals may support the establishment of a midwifery education evaluation center to implement a training-based licensing system. In the United Kingdom and Australia, where a training-based licensing system has been operated, there is a university education evaluation system that is responsible for conducting objective quality control of the midwifery curriculum [10].

### Comparison with previous studies

No comparable study was found on the current topic in literature databases, including PubMed and KoreaMed (https://koreamed.org/), because the Korean Midwifery Licensing Examination faces special conditions where the number of candidates is around 10 a year.

### Limitations

The questionnaire was prepared with neutral terms to minimize potential bias on the research topic. However, potential bias may remain.

### Generalizability

This study’s results are difficult to apply to midwifery license examinations outside Korea. The issues dealt with in this study are unique to Korea.

### Suggestions for further studies

A follow-up study is recommended for an efficient action plan for operating midwifery licensing examinations with few candidates.

### Conclusion

This study compared and analyzed the examination-based licensing system and the training-based licensing system to provide a more efficient plan for midwifery licenses in Korea according to changes in childbirth medical infrastructure. Agreement was reached between and within occupations regarding license acquisition for midwives. Maintenance of the examination-based licensing system is still essential for midwives; the training-based licensing system should be considered and discussed given questions regarding the efficiency of having a licensing examination for a tiny number of candidates.

## Figures and Tables

**Figure f1-jeehp-20-15:**
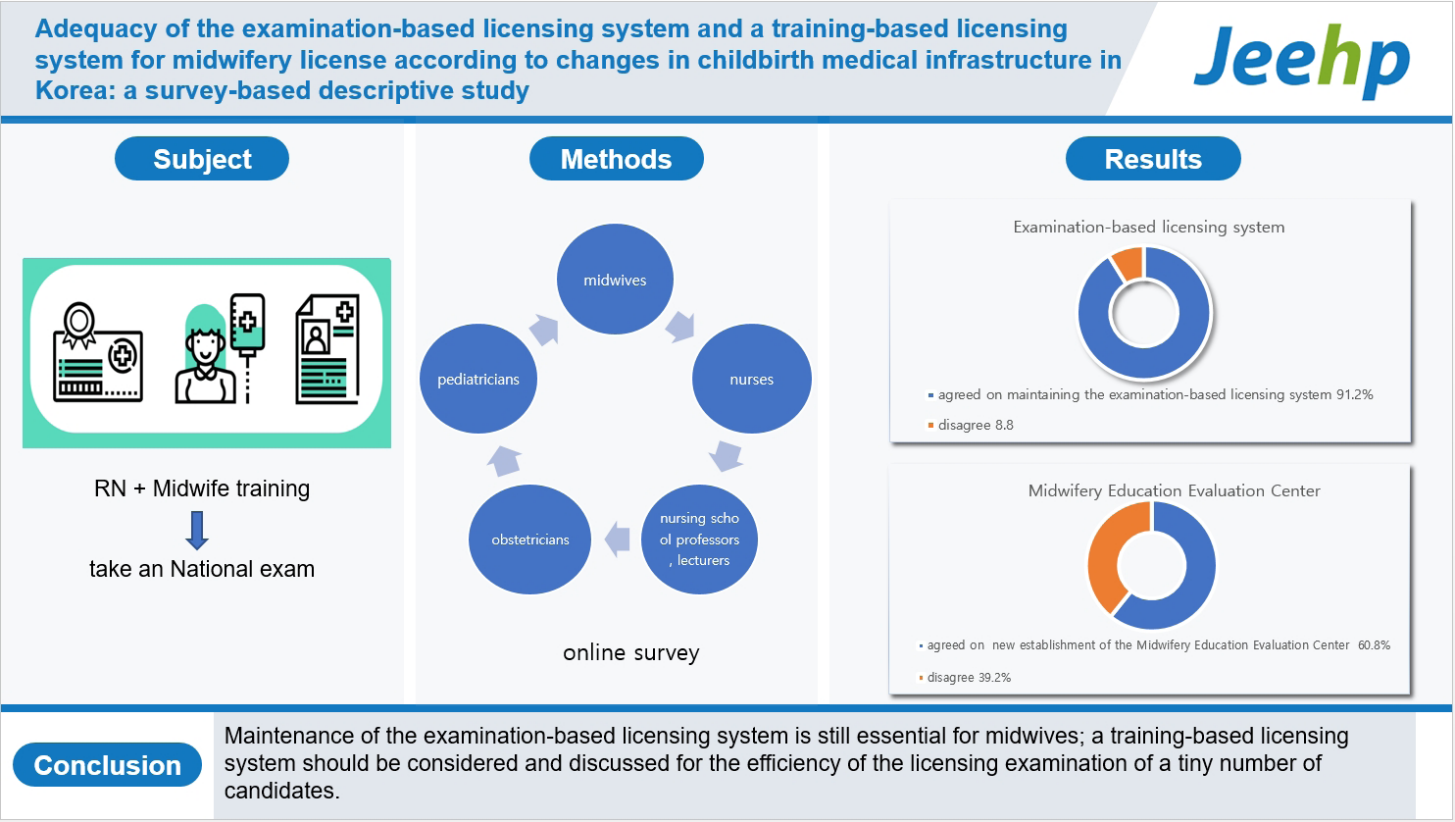


**Table 1.** General characteristics of research subjects for the midwifery license system (N=217)

Characteristic	Value
Age (yr)	48.11±10.56 (26–76)
<30	6 (2.8)
30 to <40	42 (19.4)
40 to <50	68 (31.3)
50 to <60	70 (32.2)
≥60	31 (14.3)
Gender	
Female	211 (97.2)
Male	6 (2.8)
Marital status	
Married	168 (77.4)
Single	49 (22.6)
Level of education	
3-Year’s degree	25 (11.5)
Bachelor’s degree	95 (43.8)
Master’s degree	49 (22.6)
Doctoral degree	48 (22.1)
Midwife license	
Yes	126 (58.1)
No	91 (41.9)
Occupation	
Midwife	53 (24.4)
Nurse	73 (33.6)
Nursing school professor and instructor	48 (22.1)
Obstetrician	15 (6.9)
Pediatrician	6 (2.8)
Other^a)^	22 (10.1)
Place of employment	
Hospital	126 (58.1)
Maternity clinic	6 (2.7)
University	52 (24.0)
Public health center	11 (5.1)
Other	22 (10.1)
Obstetrics/pediatrics	12.49±9.97
None	14 (11.3)
Total working experience (yr)^b)^	0–40.0
≤5	27 (21.4)
>5 to 9	20 (15.9)
10 to 14	17 (13.5)
15 to 19	17 (13.5)
20 to 24	15 (11.9)
25 to 29	11 (8.7)
≥30	5 (4.0)

Values are presented as mean±SD (range), number of individuals (%), mean±SD, or range. SD, standard deviation.

a) Other occupations included public officials, school health instructors, office workers, those on leave of absence, and unemployed, including retirees.

b) Applicable only to midwives and nurses.

**Table 2.** Survey on the licensing system for midwifery in Korea (N=217)

Question	All (N=217)	Midwife (n=53)	Nurse (n=73)	Nursing school professor/instructor (n=48)	Obstetrician (n=15)	Pediatrician (n=6)	Other (n=22)
I agree with maintaining the existing national licensure examination system.							
Yes	198 (91.2)	49 (92.5)	65 (89.0)	44 (91.7)	15 (100.0)	6 (100.0)	19 (86.4)
No	19 (8.8)	4 (7.5)	8 (11.0)	4 (8.3)	0	0	3 (13.6)
I agree with implementing a training-based licensing system to cover the examination costs due to the decreasing number of applicants.							
Yes	94 (43.3)	24 (45.3)	34 (46.6)	15 (31.3)	10 (66.7)	0	11 (50.0)
No	123 (56.7)	29 (54.7)	39 (53.4)	33 (68.8)	5 (33.3)	6 (100.0)	11 (50.0)
I agree with establishing a midwifery education evaluation center for the training-based licensing system in Korea.							
Yes	132 (60.8)	33 (62.3)	46 (63.0)	28 (58.3)	11 (73.3)	3 (50.0)	11 (50.0)
No	85 (39.2)	20 (37.7)	27 (37.0)	20 (41.7)	4 (26.7)	3 (50.0)	11 (50.0)
What would happen to the quality of midwifery if midwives were produced only by a training-based licensing system?							
No difference	40 (18.4)	6 (11.3)	13 (17.8)	6 (12.5)	8 (53.3)	0	7 (31.8)
Lower	163 (75.1)	42 (79.3)	55 (75.3)	39 (81.3)	6 (40.0)	6 (100.0)	15 (68.2)
Higher	14 (6.5)	5 (9.4)	5 (6.8)	3 (6.3)	1 (6.7)	0	0
Should the training of midwives as birth support personnel be promoted in Korea?							
Yes	197 (90.8)	50 (94.3)	62 (84.9)	43 (89.6)	14 (93.3)	6 (100.0)	22 (100.0)
No	20 (9.2)	3 (5.7)	11 (15.1)	5 (10.4)	1 (6.7)	0	0

Values are presented as number (%).

## Footnotes

**Data availability**
Data files are available from Harvard Dataverse: https://doi.org/10.7910/DVN/HYETXY Dataset 1. Raw response data for comparing the examination-based licensing system and the training-based licensing system for midwives.

## Supplementary materials

Supplementary files are available from Harvard Dataverse: https://doi.org/10.7910/DVN/HYETXY

Supplement 1. Questionnaire items on the examination-based licensing system and a training-based licensing system.

Supplement 2. Audio recording of the abstract.
